# Innovative design and modelling to improve sex and gender analysis in clinical trials

**DOI:** 10.1136/bmj-2025-085681

**Published:** 2025-10-10

**Authors:** Sanne A E Peters, Bronwyn M Graham, Otavio Berwanger, Katie Harris, Mark Woodward, Jane E Hirst

**Affiliations:** 1 The George Institute for Global Health, School of Public Health, Imperial College London, London, UK; 2 Julius Center for Health Sciences and Primary Care, University Medical Center Utrecht, Utrecht, the Netherlands; 3 The George Institute for Global Health, UNSW Sydney, NSW, Australia; 4 School of Psychology, UNSW Sydney, Australia

## Abstract

**Jane Hirst and colleagues** argue that novel modelling approaches using routinely collected data can be only as representative and complete as the original data, and that bridging the sex and gender gap through contemporary, innovative clinical trial designs could be a crucial way forward

Although women represent half of the world’s population, they comprise fewer than half of the participants in clinical trials. This has created gaps in knowledge about women’s health, as well as how sex and gender affect health outcomes for everyone.^1^ For this article, part of the BMJ Collection on Women’s Health Innovation (www.bmj.com/collections/womens-health-innovation), we use definitions of sex and gender as outlined by the MESSAGE project (box 1).^2^ Historically, self-reported gender has been missing from data collection, with sex captured in a binary manner.^3^ Ultimately, we view the solutions presented in this article as components of a broader toolkit necessary to begin to represent the complexity of sex and gender in data collection, analysis, and interpretation to influence clinical practice. However, we recognise that modifications or expansions of the toolkit may be needed to ensure adequate consideration of the broader sociocultural constructs relevant to gender, sex and gender interactions, and other intersectional factors.

Box 1 **Definitions of sex and gender^2^**

The terms sex and gender are understood differently in different contexts, societies, groups, and languages. The use of these terms has changed over time and will continue to evolve.

- In biomedical research contexts, sex refers to biological attributes that differentiate female and male people (including chromosomes, hormones, and sex organs). Although typically classed as female or male, some people are born with natural variations in sex characteristics that do not fit the medical norms of female or male bodies
- Gender refers to a person’s identity, behaviour, experiences, social norms, and power dynamics. An individual’s gender may exist on a spectrum and can change over time
- Sex and gender can be operationalised categorically or dimensionally, and the chosen method must be justified according to its relevance to the question of interest
- Sex and gender interact with other factors such as age, ethnicity, socioeconomic status, and sexual orientation. Sex and gender affect people in different ways owing to the compounding effects of these other variables. Research that is motivated by the promotion of social justice and investigates how sex and gender are mutually shaped with these other variables, as well as geopolitical, colonial, and cultural forces for power and oppression, is termed “intersectional”

The consequences of gaps in knowledge about sex and gender include misdiagnosis (for example, in cardiovascular disease,^4^ attention deficit-hyperactivity disorder,^5^ and multiple sclerosis^6^), drug misdosing (for example, in the absence of sex specific treatment guidelines, unnecessarily high doses of drugs for heart failure cause increased adverse effects in women^7^), and inappropriate treatments (for example, under-prescription of drugs for pain conditions for women^8^). Lack of diversity in clinical trials leads to uncertainty surrounding the effects of therapies in people from different genders or racial and ethnic groups, at different life stages, and living in different contexts.

Over the past three decades, many policies on sex and gender integration in research have evolved.^9^ However, the under-representation of women in clinical trials remains a contemporary challenge, with women comprising approximately 40% of phase 1-3 clinical trials between 2016 and 2019, even for health conditions that are more prevalent among women than among men, such as psychiatric illness.^10^ Likewise, women are typically under-represented in trials of many conditions that are their biggest killers, including cardiovascular disease, dementia, and stroke.^11^ ^12^ ^13^ Health data on trans and gender diverse people are severely lacking.

The way data are collected is not just a problem of representation but a failure of innovation. Innovation in trial design, analysis, and regulation should help to generate more inclusive and relevant evidence. Current systems prioritise simplicity, speed, and average effects over equity and real world relevance. If we want medical research to work for everyone, we must rethink how we design trials and who we design them for. Innovative data modelling and trial designs are those that challenge the traditional parallel, double blind, placebo controlled, randomised controlled trial. Randomised controlled trials are increasingly being conducted by for-profit organisations at great expense, in contexts and populations far removed from the patients in whom the intervention will be used. We present examples of alternative approaches that we believe will help to strengthen causal inferences for sex and gender and close the gender trial gap.

## Problem with traditional randomised controlled trials

Funders and regulatory requirements increasingly encourage or mandate equal representation of female and male participants, although this has frequently not been achieved in practice. Randomised controlled trials often fail to report subgroup analyses by sex, leaving no indication of potential sex differences in efficacy and safety outcomes.^14^ Even if no sex difference is apparent, sex specific results (including confidence intervals) should still be reported, as a lack of detectable difference may stem from too few participants of one or both sexes. Consideration should also be given to early stopping of trials if evidence of harm is observed within one of the study subgroups (for example, where a signal of harm is apparent in one sex in a drug trial).^15^

Detecting sex specific effects of a drug or intervention with adequate precision usually requires a much larger sample size (perhaps twice as large) than in a traditional randomised controlled trial, which is often impractical.^16^ However, doubling the sample size is unlikely to provide enough power to detect meaningful sex differences in effects, rather than just precise sex specific effects. Meta-analyses can overcome this power problem if sex specific results are available. Better still, meta-analyses using individual patient data can allow researchers to examine effects not only by sex but also by how sex interacts with other factors such as ethnicity, disability, and income. Unfortunately, accessing individual patient data for meta-analyses poses practical challenges; for example, the Blood Pressure Lowering Treatment Trialists’ Collaboration identified 100 studies eligible for participation but obtained individual patient data from only 51.^17^ ^18^ Policies that promote sharing of individual patient data from trials would be highly beneficial.

## Modelling observational data to help to close trial sex and gender gaps

Generating evidence is not limited to trial design; it also includes innovative uses of real world data and advanced modelling techniques. With the increasing availability of routine care data and other large scale studies, modelling approaches using observational data can overcome sample size limitations and answer questions when randomised controlled trials would be unethical or unfeasible. These include studies to determine optimal drug regimens by sex that can also include broader considerations of intersectionality and non-binary aspects of sex and gender. We highlight three innovative approaches using observational data that strengthen causal inference and could help to close persistent evidence gaps related to sex and gender in clinical research.

### Target trial emulation

Target trial emulation is a systematic approach that closely mimics the design and outcomes of traditional randomised controlled trials as closely as the data permit, using existing observational data.^19^ It involves the detailed specification of the hypothetical randomised controlled trial protocol, including eligibility criteria, treatment strategies, follow-up period, outcomes, and data analysis plan. Once the protocol has been defined, each component should be emulated as accurately as possible using observational data. If successful, the emulation yields effect estimates similar to those of the target trial. Some limitations of target trial emulation include its dependence on data from clinical practice, which means that it cannot be used to evaluate new treatments or compare them against a placebo. When considering sex and gender, often only sex will be available. Target trial emulation can also only be done in health systems with comprehensive electronic medical record systems and in settings with trained researchers. This has resulted in a global disparity in application, with only 6% of published target trial emulations originating from low and middle income countries.^20^

Although target trial emulation can help in assessing the applicability of randomised controlled trial findings to broader populations than those included in the trial, it has not yet been widely applied to examine sex specific treatment effects. An exception is a Danish registry study that assessed the risk of major adverse cardiovascular events associated with diclofenac. In this study, cardiovascular events were higher among female than male participants who initiated diclofenac.^21^ Although this information has not, to our knowledge, yet changed clinical practice guidelines, it serves as an example of how this approach could be applied.

### Digital twins

The concept of digital twins is gaining increasing attention in healthcare. A digital twin is a virtual representation of a person, often built using machine learning and multiscale modelling, which can be used for various applications, including the monitoring and prediction of health trajectories or the simulation of potential treatment strategies.^22^ Recently, digital twins have also been used to evaluate the generalisability of trial results and to translate evidence across patient populations. For example, using data from two trials with discrepant results on the effect of intensive blood pressure control on cardiovascular outcomes, a digital twin approach that conditioned each randomised controlled trial on the other randomised controlled trial’s patient population reproduced the treatment effects in the trials.^23^ The approach was also applied to real world populations, showing the potential to extend inferences to broader patient populations. Digital twins have been proposed to serve as computational models of female physiology, including pregnancy and hormonal cycles,^24^ the female pelvic floor,^25^ and brain changes during the menstrual cycle.^26^ Such models could provide novel avenues for testing the effects of interventions in female specific or female dominant conditions.

These applications are promising, but an essential limitation of existing digital patient twins is that they are often trained on datasets in predominantly white male and affluent populations and fail to incorporate gender sensitive and other intersectional factors, thereby reinforcing existing health disparities and limiting clinical effectiveness.^27^ Hence, to broaden the application to under-represented groups, these biases must be overcome to close, rather than exacerbate, existing data gaps.

### Drug target mendelian randomisation

Mendelian randomisation is sometimes described as a “natural” randomised trial. At the moment of conception, we each inherit a random mix of genetic variants from our parents. Mendelian randomisation takes advantage of this natural randomisation to test whether certain factors—such as body mass index—actually cause particular health outcomes.^28^

Mendelian randomisation can shed light on possible differences between men and women. For example, observational studies have long shown that diabetes is more strongly associated with the risk of coronary heart disease in women than in men. When researchers used sex specific mendelian randomisation, however, they found no genetic evidence for such a difference.^29^ By contrast, mendelian randomisation has shown that body mass index related genetic risk for diabetes is stronger in women than in men.^30^ A newer twist on the method, known as drug target mendelian randomisation, uses genetic variants near the gene for a drug’s biological target to mimic the effect of that drug. These variants can influence the relevant protein, the amount of the protein produced, or a biomarker that the drug affects. Although few studies have looked at drug target mendelian randomisation by sex, some have already shown differences—for example, in drug targets for heart failure.^31^ Extending this approach to more conditions, including those never tested in clinical trials, could help to uncover important sex specific effects of treatments.^32^

## Innovative clinical trial design to close the sex and gender gap

Although modelling can sometimes help in understanding sex differences, these methods face several challenges, including the difficulty of accessing high quality longitudinal data, the need to comply with data governance and regulatory standards, high costs associated with acquiring the data, and the need for advanced statistical and computational skills. Prospective clinical trials with enough participants from diverse backgrounds, including different sexes and genders, that accurately represent the target population for the intervention being studied are still needed. Achieving this will demand greater diversity in the leadership of clinical trials,^33^ as well as innovative approaches to design, recruitment, and retention of participants. We outline three design strategies aimed at correcting the under-representation of women in randomised controlled trials.

### Strategy 1: streamlining (simplifying) eligibility criteria by minimising reasons for exclusion

Many trials use complex screening and overly stringent eligibility criteria, thereby excluding large groups of people who might benefit from the intervention.^34^ This leads to study populations that do not reflect real world patients. Simplifying eligibility criteria can help to overcome this problem. Exclusion criteria without valid scientific justification—especially those based on female reproduction—should be avoided. For example, an analysis of 283 randomised trials found that nearly 40% included unjustified exclusions related to pregnancy.^35^ In the UK, 1.1% of clinical trials allow the inclusion of pregnant women and 0.6% allow lactating women.^36^ Although some of these exclusions will be necessary to protect the fetus, this is not always the case, as was illustrated during the testing of covid-19 vaccines.^37^ These exclusions mean that we have very little evidence to guide the medical management of women who are pregnant or lactating.

### Strategy 2: pragmatic trial models using decentralised features and routinely collected data

Decentralised trials aim to “meet patients where they are.” This includes methods such as screening and recruitment through online identification, drug delivery to patients’ homes, home based testing, virtual follow-up via telemedicine, and data collection through secure study web portals and mobile apps. Decentralised trials can help to overcome barriers to participation that may explain lower involvement among women—for example, by reducing travel and time commitments for people with responsibilities such as childcare and care giving.^38^ In settings where routinely collected data are not easily accessible or where access to digital technology is limited, alternative decentralised methods—such as mobile clinical trial units or community based recruitment facilitated by healthcare workers—can be used.^39^ This is especially important for low and middle income countries, where electronic data collection platforms with both online and offline capabilities are highly advantageous. Community based, digital data capture has been successfully used in female specific conditions (for example, pregnancy and cervical cancer); however, fewer examples exist of decentralised approaches promoting equitable recruitment in conditions affecting both sexes. The SAPPHIRE-LDL trial, which is ongoing in Brazil, is investigating a digital strategy to support cholesterol management in people with atherosclerotic cardiovascular disease and illustrates several decentralised elements, including the use of digital technologies. The trial investigators have published the baseline characteristics of their participants, with 40% female participants and diversity in representation of race, income, and educational levels.^40^ Overall, however, the application of decentralised approaches to date has remained limited in low and middle income countries, probably owing to technical barriers, regulations, ethics, and contextual and social acceptability.^41^

The second part of a decentralised approach is the use of routinely collected data or national registries to facilitate rapid and efficient online patient screening and recruitment of both female and male participants. The Scottish SHARE database, established in 2012, has more than 300 000 people over the age of 11 years registered.^42^ This platform identified women in the community with heavy menstrual bleeding for an early phase trial of oral low dose dexamethasone to reduce bleeding.^43^ This shows the potential for platforms such as SHARE for increasing trial participation. Routinely collected data can also facilitate trial follow-up, decreasing the burden on participants and research teams, and decreasing dropout and discontinuation rates^44^; however, this approach is reliant on the availability of high quality, interoperable, and secure electronic medical record systems. This will be limited in countries and contexts that lack government support for interoperability or standardised health datasets.

### Strategy 3: patient and public involvement

The involvement of people with lived experience and their families, carers, and community in the co-development and conduct of clinical trials is a priority. Genuine co-design and engagement with people who have lived experience have the added advantage of expanding trial access, and recruitment and retention rates are improved.^45^ Without understanding the lived experiences of women, men, and people of other genders, redesigning trials to meet their needs will not be possible. This approach has been shown to be effective in improving representation and engagement with women and other underserved groups.^46^

## Recommendations for innovative approaches to closing the sex and gender gap in clinical trials

Sex and gender bias in clinical trials remains a significant challenge and compromises the evidence needed for informed health decision making. We highlight that although advances in the use of routinely collected data, multimodal digital twins, and target trial emulation could provide novel insights beyond what is possible through traditional randomised controlled trials, these approaches are ultimately limited by the quality, completeness, and representativeness of the data on which they are built. Action will need the commitment, resourcing, and training of all actors involved in the research and development pathway, with a focus on global equity (table 1). Some barriers to implementation, such as regulatory barriers, access, and cost, require action at the national and multilateral levels. Ultimately, a combination of innovative approaches to clinical trial design and novel approaches to data and modelling could help to bridge the sex and gender gap and provide high quality evidence to inform treatment practices in both female and male patients, and across the sex and gender spectrum.

**Table 1:** Recommendations to close sex and gender gap in clinical trials

Who?	What?	How?
Medicine and device regulatory bodies	Incentivise trials that are designed and powered to answer questions around sex and gender differences in treatment response, side effects, and access	Incentives such as tax credits and priority review vouchers
		Consider how modelled data can complement data from RCTs where sample size would be prohibitive
		Mandate sex disaggregated presentation of data for regulatory approval*
		Require incorporation of sex and gender effects on all medication inserts*
Clinical triallists from academia and industry	Greater representation of women, gender diverse, and trans people in clinical trials	Adopt decentralised and pragmatic designs
		Work closely with public and patient representatives to design trials with minimal burden on participants*
AI modellers and data scientists	Develop modelling approaches such as digital twins that can account for sex specific human anatomy and physiology, including hormonal cyclic effects, pregnancy, menopause, and exogenous hormone use in trans people	Identify multimodal data (eg, imaging, data from apps tracking menstrual or menopausal symptoms trackers, smart watches, and other wearables) to add to existing modelled data from clinical trials or routinely collected medical record data
Patient and public groups	Advocate for disaggregated data and sex and gender specific guidelines where needed	Raise awareness of the importance of sex and gender specific approaches to care through mainstream and social media campaigns and lobbying for further investment.
Funders of clinical research (government, philanthropic, industry)	Adopt standardised terminology and rules around how sex and gender are accounted for at all levels of research	Support national frameworks to guide sex and gender considerations in medical research (eg, MESSAGE project UK)*
		Provide training to applicants and reviewers on how to assess whether accounting for sex and gender is appropriately justified*
Medical journal editors	Enforce guidance such as SAGER for publication. Mandate data be made available on request or open access and be sex disaggregated	Adoption of an agreed code of conduct across the major publishing houses, publicised widely to academic institutions, CROs, and pharmacological research companies with an agreed date of global implementation

AI=artificial intelligence; CRO=contract research organisation; RCT=randomised controlled trial.

* These are the most urgent and feasible recommendations for resource constrained settings.

**Key messages**

Women remain under-represented in clinical trials in many disease areas
Sex or gender (where relevant) specific data should be reported for all trials to facilitate meta-analysis and provide appropriate information to guide women’s healthcare
Modelling and statistical approaches can be applied to traditional trial and observational datasets to weight data, emulate clinical trials, or create digital twins; however, the inferences from these methods will be limited by the quality and quantity of the source data
Innovative clinical trial designs, including decentralised trials and streamlined eligibility criteria, and greater involvement of the public and patients at all stages of the research are tools to improve inclusion across the sex and gender spectrum in future trials

## Notes

Contributors and sources: The authors bring expertise in women’s health (JEH), epidemiology, statistics and sex differences in epidemiology and clinical trials (SAEP, KH, MW), innovative clinical trials (OB), and sex and gender differences in medical research (BG, MW). SAEP is an epidemiologist who has investigated sex differences across a range of non-communicable diseases and is an associate professor at the University of Utrecht, the Netherlands, and Imperial College London. JEH is director for the George Institute for Global Health Women’s Health Program and chair of Global Women’s Health, Imperial College London. BG is the director of the Centre for Sex and Gender Equity in Health and Medicine in Australia. OB is the chair of clinical trials, Imperial College London, and executive director of the George Institute for Global Health, UK. KH is a statistician working on sex differences in several health conditions. MW is chair of statistics, epidemiology and women’s health and professor of medical statistics and leads an extensive programme of work on sex differences in health outcomes. All co-authors contributed equally to the development of the concept and writing of this article, drawing on their expertise in their respective fields.

This article is part of a collection developed in partnership with the Gates Foundation. Open access fees were funded by the Gates Foundation. *The BMJ* commissioned, peer reviewed, edited, and made the decision to publish. Smruti Patel and Jocalyn Clark were the lead editors for *The BMJ*.
